# Health Records of 1918

**Published:** 1920-01-10

**Authors:** 


### Health Records of 1918.

Dr. Hamer's public health report for 1918 has been issued at the moderate price of 1s. 9d. The first point noted is that for the first time in the records, which now go back nearly a century, deaths exceeded births. The main causes of this phenomenon were, of course, two—influenza on the one hand and the war on the other. The population of the capital city is estimated as less than in 1914, less than in 1911 too, despite the fact that London, as the centre of military administration, had a smaller relative decrease than England and Wales as a whole. With the freedom from air raids a reaction set in, however, and Dr. Hamer thinks that, judging from the existing demand for accommodation, there must be as many people in London as there were in 1914. The rise in the marriage rate is largely attributable to enlistments. Decline in births bears most, it is surprising to learn, upon the poorer class districts, such as Stepney, Bethnal Green, and Shoreditch, births there being as much as 10 per cent. fewer than in Hampstead, Lewisham, and Wandsworth. Deaths from tuberculosis were more, as is well known. In disagreement with experience from several sources, Dr. Hamer attributes this in part to the influenza epidemics. Extra-pulmonary tubercle, it is gratifying to note, continues to diminish, especially in young children. There is an interesting criticism made on a recent investigation into the epidemiology of phthisis. Dr. Brownlee, it will be remembered, promulgated the view that there are several types of this disease, affecting several age periods of life, a "young adult" type, a "middle-age" group, an "old-age" variety, and so on. Dr. Hamer points out that in this investigation no mention is made of the effects of movements of population upon the phthisis mortality at different ages. As an instance he takes the Cornish tin miners, who emigrate in great numbers to work in other parts of the world as soon as they have learned their craft, between the ages of twenty and twenty-five, returning at about sixty-five (not necessarily to the same district) to retire from work, as shown by the Registrar-General's occupational statistics for 1900-2. The phthisis mortality of Cornwall is thus based upon a residual population, and as such is fallacious, in particular fallacious as regards Dr. Brownlee's Cornish "old-age" phthisis. The venereal disease clinics at various general hospitals did a considerable amount of work, treating over 16,000 patients; it has to be remembered, though, that general hospitals have a fair number of these patients in any case. The report concludes with an epidemiological examination of the two great outbursts of influenza during the year, an investigation which is marked by wide historical knowledge of epidemic disease. The congregation of large numbers in camps or munition factories is absolved from the principal share in promoting infection, although it is a factor to be taken into account. In general the disease behaved freakishly, showing everywhere exceptional features which prevented any broad deductions being drawn. However, in the summer epidemic the poorer class suffered proportionately much more than in the autumn, the conclusion being drawn that from immunity production the class scourged in the summer was somewhat spared later on in the year. Dr. Hamer thinks that the disease remains endemic in large centres of population; but that in thinly populated areas, as in Central Asia and the New World, it may be absent for a century, so that when introduced into a highly susceptible community it gathers strength quickly, and in these days of rapid wide communication becomes carried all over the earth. The problem of influenza thus remains unsolved. Happily, war conditions are less with us now to aggravate its urgency.

